# Nutritional Status Assessment Using the Patient-Generated Subjective Global Assessment (PG-SGA) in Individuals with Colorectal Cancer Undergoing Chemotherapy Regimens

**DOI:** 10.3390/jcm14186664

**Published:** 2025-09-22

**Authors:** Luis Enrique Sánchez-Diestro, Raquel Macias-Montero, Ana Isabel Ramalho-Galhanas, Ana Maria Aguiar-Frias, María Sandra Paniagua-Vivas, Jorge Guerrero-Martín

**Affiliations:** 1 Department of Nursing, Faculty of Medicine and Health Sciences, University of Extremadura, 06071 Badajoz, Spain; jorguerr@unex.es; 2 Department of Biomedical Sciences, Faculty of Medicine and Health Sciences, University of Extremadura, 06071 Badajoz, Spain; raquel.macias@salud-juntaex.es; 3 Department of Medical Oncology, University Hospital Complex of Badajoz, Extremadura Health Service, 06080 Badajoz, Spain; 4 Local Health Unit of Central Alentejo, 7000-811 Évora, Portugal; anaisabelgalhanas@gmail.com; 5 Department of Nursing, São João de Deus Higher School of Nursing, University of Évora, 7004-516 Évora, Portugal; anafrias@uevora.pt; 6 Comprehensive Health Research Centre (CHRC), University of Évora, 7004-516 Évora, Portugal; 7 Department of Nursing, University Centre of Mérida, University of Extremadura, 06800 Mérida, Spain; mariasanpv@unex.es

**Keywords:** nutrition, colorectal cancer, Folfox, Xelox, nutritional status, functional capacity

## Abstract

**Background/Objectives:** Colorectal cancer (CRC) has high prevalence and mortality, with a high frequency of malnutrition during chemotherapy (60–70%). Malnutrition reduces treatment tolerance, quality of life, and survival. The Patient-Generated Subjective Global Assessment (PG-SGA) is effective in detecting it, but its use is not systematic. This study aims to analyze the prevalence and degrees of malnutrition in CRC patients undergoing active chemotherapy, using the PG-SGA, and to propose its systematic implementation in oncology care protocols. **Methods:** Observational, cross-sectional, and descriptive study in patients with stage III–IV CRC receiving FOLFOX, XELOX, or irinotecan regimens. Nutritional status was assessed with PG-SGA and body composition by bioimpedance. **Results:** The study includes 91 patients. A total of 45.05% of patients required intensive nutritional intervention, 39.56% needed dietary and pharmacological measures, 10.99% required health education, and only 4.4% did not require intervention. FOLFOX was significantly associated with taste alterations (*p* < 0.001), nausea (*p* = 0.020), unpleasant odors, and eating problems; XELOX with diarrhea (*p* = 0.009) and xerostomia (*p* = 0.038). Irinotecan was related to poorer functional capacity (*p* = 0.042). Oxaliplatin was linked to loss of appetite (*p* = 0.034) and unpleasant odors (*p* = 0.035). Older age correlated with a greater need for intensive interventions. **Conclusions:** The study shows a high nutritional risk in oncology patients, particularly in those with colorectal cancer undergoing FOLFOX treatment, associated with symptoms that impair intake and functional capacity. The systematic implementation of nutritional screening from the onset of treatment, using tools such as the PG-SGA and GLIM criteria, is essential for early detection and individualized management, improving therapy tolerance, clinical outcomes, and quality of life.

## 1. Introduction

Colorectal cancer (CRC) is one of the leading causes of incidence and mortality from neoplasms worldwide, with a growing clinical and socioeconomic impact [1]. In Western countries, it represents one of the most prevalent digestive tumors, ranking among the three most frequently diagnosed cancers in both men and women [2]. Its high clinical burden is associated not only with the oncological process itself but also with treatment-related complications, among which disease-associated malnutrition stands out [3,4]. Moreover, alterations in body composition, such as reduced muscle mass or changes in fat distribution, have been shown to significantly influence postoperative morbidity and oncologic survival in patients with non-metastatic colon cancer, highlighting the importance of nutritional status as a prognostic factor [5].

Malnutrition in patients with cancer is recognized as a negative prognostic factor that increases treatment toxicity, reduces overall survival, and significantly deteriorates quality of life [6]. In the specific context of CRC, the prevalence of malnutrition may exceed 60–70% during active chemotherapy treatment, due to the interaction between systemic inflammatory status, increased metabolic demands, and the onset of symptoms that limit food intake [7]. These symptoms include anorexia, dysgeusia, nausea, early satiety, diarrhea, and abdominal pain, all of which are common in patients undergoing intensive chemotherapy regimens [8].

In this regard, certain commonly used chemotherapy regimens, such as FOLFOX (5-fluorouracil, folinic acid, and oxaliplatin), have been associated with a higher incidence of gastrointestinal toxicities, dysgeusia, mucositis, and neuropathies that affect patient functionality and eating patterns [9]. XELOX (capecitabine and oxaliplatin), on the other hand, tends to present a somewhat more tolerable toxicity profile in terms of nausea and asthenia, which may have a less severe impact on nutritional intake [10]. The use of irinotecan, particularly in regimens such as FOLFIRI, has been linked to a high frequency of severe diarrhea and functional decline, contributing to nutritional deterioration and sarcopenia [11].

Given this clinical scenario, early identification of nutritional risk is essential. The Patient-Generated Subjective Global Assessment (PG-SGA) is a validated tool that allows for a comprehensive evaluation of nutritional status through key clinical variables such as weight loss, changes in intake, gastrointestinal symptoms, and functional capacity [12]. Its use has proven effective in detecting malnutrition in patients with cancer and facilitates the implementation of early dietary interventions tailored to the type of oncological treatment [13,14].

Indeed, personalized nutritional interventions, combining dietary counseling, specific meal plans, and oral supplements, have demonstrated significant improvements in weight, muscle mass, and reductions in PG-SGA scores in patients with gastric and colon cancer, highlighting the clinical value of routinely integrating this approach into oncological practice [15].

Despite its usefulness, the PG-SGA is not systematically used in many hospital centers in Spain as part of the standard approach for patients with CRC, where current guidelines do not mandate routine nutritional screening. Its formal integration into clinical protocols could allow for more precise nutritional screening and improve the clinical and functional outcomes of patients undergoing active treatment.

This study aims to assess the prevalence and severity of malnutrition in patients with colorectal cancer undergoing active chemotherapy, to explore potential differences in nutritional risk between treatment regimens (FOLFOX vs. XELOX), and to evaluate the applicability of standardized nutritional screening and assessment tools (PG-SGA and GLIM criteria) for their systematic incorporation into oncological care protocols.

## 2. Materials and Methods

### 2.1. Participants and Data Sources

An observational, cross-sectional, and descriptive study was conducted in the Medical Oncology Department of the University Hospital of Badajoz (Spain). Data collection took place between 6 November 2023 and 20 June 2024 during the routine clinical follow-up of adult patients with a histological diagnosis of stage III or IV colorectal adenocarcinoma who were undergoing active chemotherapy treatment.

Subjects older than 18 years, with sufficient cognitive ability to participate in the clinical interview and who provided written informed consent, were included. Patients with severe non-neoplastic comorbidities likely to significantly alter nutritional status were excluded. The study was approved by the corresponding ethics committee and conducted in accordance with the ethical principles of the Declaration of Helsinki.

Data acquisition was performed through a structured clinical interview, thorough review of the electronic medical record, and body composition assessment by multifrequency bioelectrical impedance analysis.

### 2.2. Demographic Variables

Sociodemographic and clinical variables were collected, including age, sex, body mass index (BMI), anatomical tumor location (colon or rectum), and clinical stage, categorized exclusively as stage III or IV.

### 2.3. Treatment-Related Variables

Administered chemotherapy protocols were documented and classified into three predominant regimens: FOLFOX (5-fluorouracil, folinic acid, and oxaliplatin), XELOX (capecitabine and oxaliplatin), and irinotecan either as monotherapy or in individualized combination regimens. Therapeutic intent was also recorded: adjuvant, neoadjuvant, or palliative.

The presence of clinical symptoms that could compromise nutritional intake was evaluated, including anorexia, nausea, vomiting, dysgeusia, diarrhea, constipation, abdominal pain, and asthenia.

### 2.4. Nutritional Status Assessment

Nutritional status was assessed using the Patient-Generated Subjective Global Assessment (PG-SGA), a validated and oncology-specific instrument that combines a patient self-administered section with a complementary evaluation by the healthcare professional.

The self-administered section includes parameters such as recent weight loss, changes in food intake and dietary type, presence of symptoms interfering with eating, and functional level. The professional clinical evaluation incorporates inspection and palpation to assess muscle mass loss, subcutaneous fat atrophy, and signs of fluid retention, in addition to estimating nutritional requirements and providing a comprehensive clinical judgment of nutritional status.

The global score is interpreted as follows: a score between 0 and 1 indicates no immediate nutritional intervention is required; a range of 2 to 3 implies the need for targeted nutritional education for the patient and family, complemented by symptom-oriented pharmacological management and analytical parameters; scores between 4 and 8 reflect the indication for joint intervention by clinical nutrition and oncology specialists; and values ≥ 9 indicate a critical situation requiring optimization of symptom control and urgent nutritional and pharmacological intervention.

Additionally, body composition was assessed using multifrequency bioelectrical impedance, quantifying lean mass, fat mass, skeletal muscle mass, bone mass, and basal metabolic rate. The bioimpedance analyzer used in the study was the TANITA^®^ RD-545-sv, manufactured by TANITA EUROPE. The results were interpreted according to manufacturer-provided reference values adjusted for age and sex, and established cut-off points from the ESPEN consensus were applied to identify malnutrition and low skeletal muscle mass.

### 2.5. Statistical Analysis

Statistical analyses were conducted using JASP software (version 0.17.0.0), implemented in R. Continuous variables were described as mean ± standard deviation or median with interquartile range, depending on the normality of distribution assessed with the Kolmogorov–Smirnov test. Categorical variables were expressed as absolute frequencies and percentages.

To evaluate the association between nutritional status according to PG-SGA and the different chemotherapy regimens, chi-square test or Fisher’s exact test were applied as appropriate. Comparisons of continuous variables between groups were carried out using analysis of variance (ANOVA) or the nonparametric Kruskal–Wallis test, depending on data distribution. A statistical significance threshold was set at *p* < 0.05.

### 2.6. Ethical Considerations

Research subjects were duly informed about the objectives, procedures, risks, and potential benefits of the study, and written informed consent was obtained before inclusion. The study was conducted in accordance with the ethical principles of the Declaration of Helsinki, ensuring respect for autonomy, confidentiality, and participants’ well-being. Furthermore, the research protocol was approved by the Ethics Committee of the Health Area Management of Badajoz, in compliance with current regulations regarding the protection of human subjects in biomedical research.

## 3. Results

### 3.1. Demographic and Clinical Characteristics of the Cohort

No refusals to complete the PG-SGA questionnaire were recorded among eligible patients; therefore, all 91 recruited subjects were included in the analysis. The sample comprised 38.46% women, with an overall mean age of 64.97 ± 9.92 years and an interquartile range from 58 years (P25) to 73 years (P75). The mean age for men was 64.27 ± 10.69 years, while for women it was 66.00 ± 8.77 years. The standard deviation for the entire cohort was 9.92 years.

### 3.2. Treatments Received and Associated Nutritional Risk

Regarding therapeutic exposures, none of the patients received high-risk chemotherapy regimens such as hematopoietic stem cell transplantation or concomitant radio-chemotherapy for head and neck or esophageal neoplasms. However, a significant proportion were undergoing treatment with cytotoxic agents associated with moderate nutritional risk, most notably oxaliplatin in 73.33% of cases, irinotecan in 14.44%, and continuous infusion 5-fluorouracil in 42.22%. In the context of routine clinical practice in the Medical Oncology Department of the University Hospital of Badajoz, the FOLFOX regimen (oxaliplatin, 5-fluorouracil, and leucovorin) and XELOX regimen (capecitabine and oxaliplatin) represent the most widely used protocols for colorectal cancer treatment. In the analyzed cohort, 78 patients received one of these two regimens, with 42 treated with XELOX.

### 3.3. Nutritional Risk Stratification

**Figure 1:**
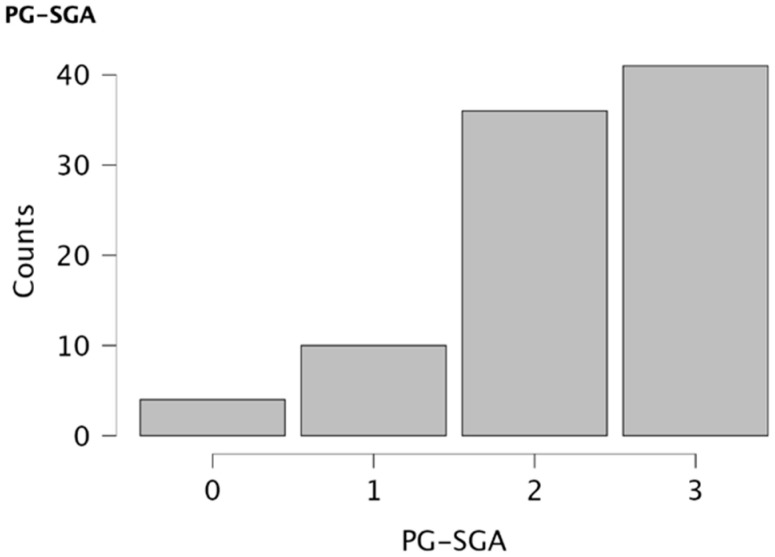
Descriptive representation of the proportion of patients who will benefit from each type of nutritional intervention. No nutritional intervention required (0), nutritional education required (1), nutritional intervention required (2), and critical need for action through recommendations for enteral or parenteral nutrition (3).

**Figure 2:**
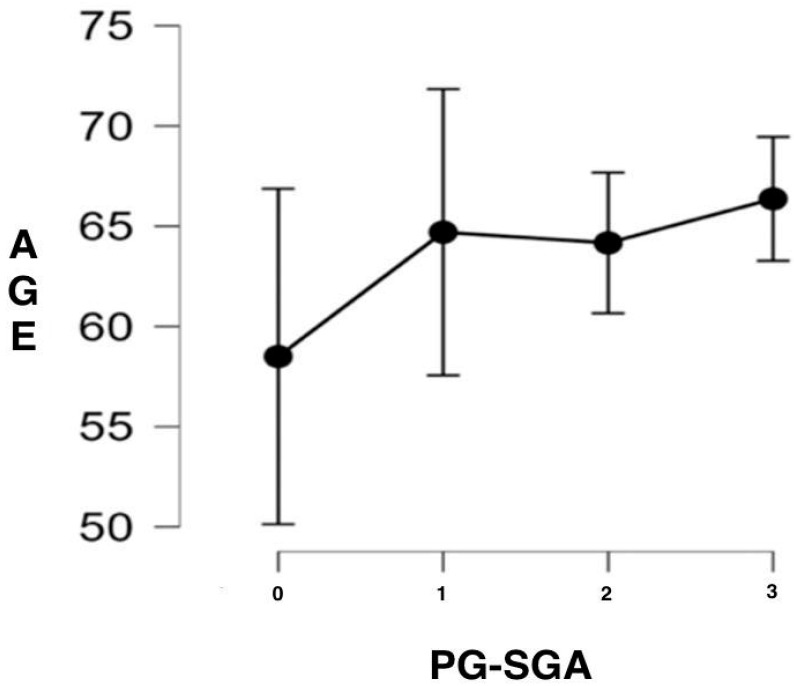
Descriptive representation of the risk presented by the patients in the study sample according to the PG-SGA in relation to their age. No nutritional intervention required (0), nutritional education required (1), nutritional intervention required (2), and critical need for action through recommendations for enteral or parenteral nutrition (3).

Analysis of PG-SGA scores (Figure 1) showed that 4.40% of patients would not require nutritional intervention, while 10.99% presented an indication for health education interventions aimed at promoting healthy dietary habits. In 39.56% of cases, pharmaceutical intervention with individualized dietary recommendations was considered necessary, including symptom management, high-protein diets, and/or oral nutritional supplements. Finally, 45.05% met criteria for the initiation of intensive nutritional intervention through enteral or parenteral nutrition. Furthermore, an increasing trend was observed in the need for more intensive interventions with advancing age (Figure 2), suggesting a possible association between aging and the severity of nutritional risk.

### 3.4. Symptomatology and Profile by Chemotherapy Regimen

The PG-SGA questionnaire differentially weighs the impact of symptoms on nutritional status. Manifestations such as diarrhea and anorexia, scored with three points on the scale, are considered more disabling for food intake and nutrient absorption than others such as constipation or xerostomia, which are scored with one point.

**Figure 3:**
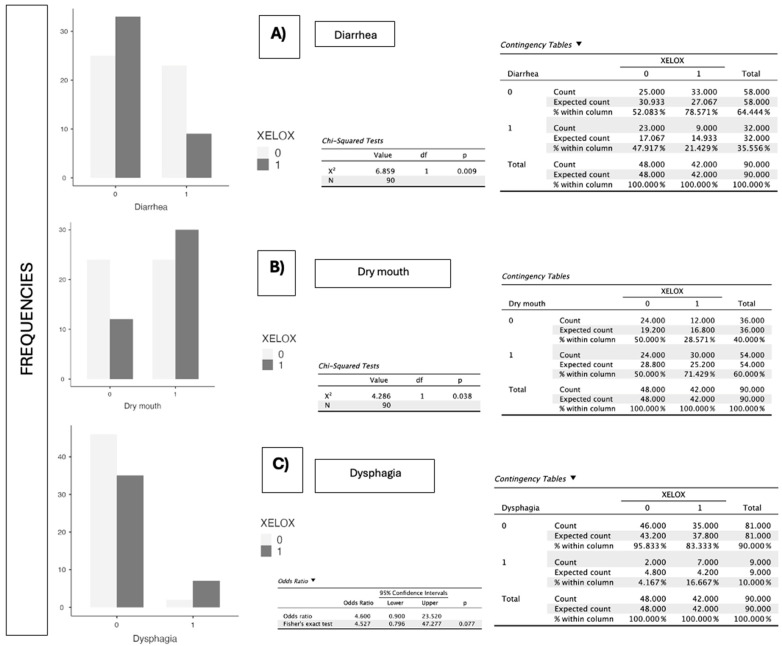
(**A**–**C**) Graphical representation of the significant difference in proportions between patients who present diarrhea, dry mouth, or swallowing difficulties and are administered XELOX, compared to those who are not administered the treatment. 0 indicates no administration and 1 indicates administration of XELOX. *p*-value obtained according to the χ^2^ test is indicated.

**Figure 4:**
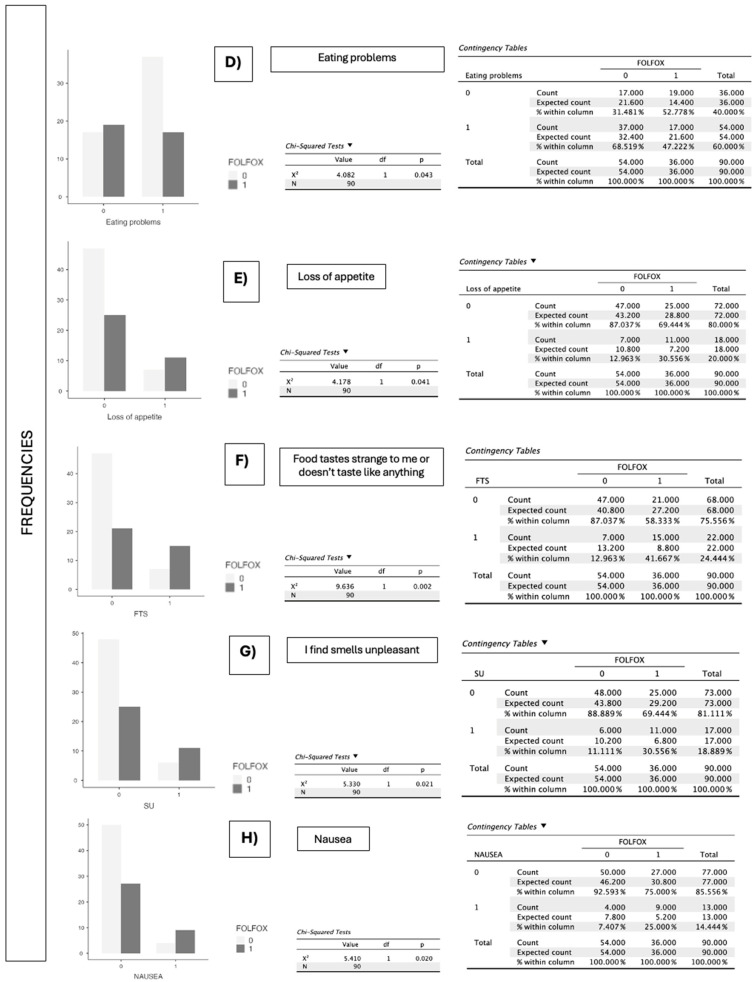
(**D**–**H**) Graphical representation of the significant difference in proportions between patients who present eating difficulties, loss of appetite, altered or absent taste perception, food aversion due to odors, or nausea, and are administered FOLFOX treatment, compared to those who are not administered the treatment. 0 indicates no administration and 1 indicates administration of FOLFOX. The *p*-value obtained according to the χ^2^ test is indicated.

In the analysis by chemotherapy regimen, XELOX (Figure 3) was significantly associated with diarrhea (*p* = 0.009) and xerostomia (*p* = 0.038), with no relevant differences found for anorexia, constipation, taste alterations, nausea, or vomiting. Conversely, FOLFOX (Figure 4) showed a statistically significant association with taste alteration (*p* = 0.002), unpleasant odors (*p* = 0.021), and nausea (*p* = 0.020), as well as a significant relationship with overall eating problems (*p* = 0.043) and loss of appetite (*p* = 0.041). No significant differences were detected for diarrhea or xerostomia in this group (Table 1).

### 3.5. Analysis by Active Principle

The disaggregation of data by active principle allowed the identification of specific associations (Table 2). In the case of oxaliplatin, statistical significance was obtained for loss of appetite (*p* = 0.034) and unpleasant odors (*p* = 0.035). The joint analysis of 5-fluorouracil and leucovorin, administered simultaneously as part of the FOLFOX regimen, showed significant associations with loss of appetite (*p* = 0.019), unpleasant odors (*p* = 0.037), overall eating difficulties (*p* = 0.037), nausea (*p* = 0.006), and taste alterations (*p* < 0.001).

With respect to functional capacity (FC), a component included in the PG-SGA scale and a determinant of quality of life in patients with cancer, the only active principle that showed a significant association was irinotecan (*p* = 0.042). No significant differences were found for FC with the rest of the drugs used in the cohort: FOLFOX (*p* = 0.433), XELOX (*p* = 0.387), oxaliplatin (*p* = 0.733), capecitabine (*p* = 0.434), 5-fluorouracil (*p* = 0.126), leucovorin (*p* = 0.141), bevacizumab (*p* = 0.139), and panitumumab (*p* = 1.000).

The PG-SGA scale classifies FC according to five progressive situations of deterioration, assigning higher scores as functional limitation increases: from absence of restrictions and maintenance of normal activity to almost complete confinement to bed. In this context, higher scores correlate with an increased nutritional risk, reinforcing the importance of FC as a complementary clinical indicator for identifying patients eligible for intensive dietetic-therapeutic intervention.

## 4. Discussion

### 4.1. Prevalence and Prognostic Relevance of Malnutrition in Colorectal Cancer

Our results confirm that malnutrition in patients with colorectal cancer (CRC) undergoing chemotherapy shows a high prevalence, in line with the literature. A recent meta-analysis determined that the combined prevalence of moderate and severe malnutrition is 47.8%, identifying chemotherapy as a significant moderating factor of nutritional risk [16]. Severe malnutrition, which reaches 19.3% in the general oncology population, is associated with poorer treatment tolerance, higher incidence of complications, and worse overall survival [17]. These data, together with our cohort findings, consolidate malnutrition as an adverse prognostic factor that should be proactively addressed from the beginning of treatment.

### 4.2. Pathophysiology and Underlying Mechanisms

The pathophysiology of chemotherapy-induced malnutrition is based on the interaction of gastrointestinal toxicity, systemic inflammation, and protein catabolism. Drugs such as 5-fluorouracil, oxaliplatin, and irinotecan cause mucositis, intestinal dysbiosis, malabsorption, and anorexia, contributing to accelerated loss of muscle mass [18,19]. Irinotecan, in particular, is linked to severe diarrhea and functional decline, accelerating progression toward cachexia and sarcopenia [9]. This adverse metabolic environment exacerbates lean mass loss and increases basal energy expenditure, compromising functional capacity and treatment response.

The comparison of chemotherapy regimens FOLFOX, XELOX, and irinotecan is relevant from a nutritional perspective due to their differences in gastrointestinal and metabolic toxicity, which affect nutrient intake, absorption, and utilization. FOLFOX commonly causes moderate diarrhea and peripheral neuropathy; XELOX may lead to diarrhea, mucositis, and liver alterations; while irinotecan is associated with more severe diarrhea and marked anorexia. These differences directly influence the risk of protein-calorie malnutrition, electrolyte loss, and vitamin deficiencies, impacting treatment tolerance, adherence, quality of life, and therapeutic outcomes. Evaluating and comparing these regimens allows for the implementation of preventive and adaptive nutritional support strategies, thereby optimizing the comprehensive management of oncology patients.

### 4.3. Influence of Age and Comorbidities

Previous studies indicate that advanced age increases nutritional vulnerability due to baseline sarcopenia, reduced physiological reserves, and the coexistence of chronic comorbidities [20,21]. These factors, together with the adverse effects of chemotherapy, explain why in our cohort patients aged 65 years and older concentrated the most intensive nutritional support needs. These factors, together with the adverse effects of chemotherapy, explain why in our cohort older patients concentrated the most intensive nutritional support needs.

### 4.4. Nutritional Screening and Diagnostic Tools

In our study, the Patient-Generated Subjective Global Assessment (PG-SGA) confirmed its value as the reference tool for nutritional evaluation in oncology [12]. Nevertheless, considering the high workload in clinical practice, tools such as the Malnutrition Screening Tool (MST) and the Malnutrition Universal Screening Tool (MUST) may represent practical alternatives, as previous studies have demonstrated their validity compared to the GLIM criteria [22,23,24]. Furthermore, the incorporation of GLIM criteria, by integrating phenotypic and etiologic parameters, appears to strengthen the predictive capacity for mortality and the identification of patients at greater nutritional risk, which is consistent with our findings [13,25,26].

Although the Malnutrition Screening Tool (MST) and the Malnutrition Universal Screening Tool (MUST) have demonstrated high validity compared to GLIM criteria and offer the advantage of rapid application in high-workload clinical settings, the PG-SGA provides a more comprehensive evaluation of nutritional status. Unlike MST and MUST, PG-SGA integrates detailed information on recent weight loss, changes in dietary intake, nutrition-impact symptoms (such as nausea, diarrhea, or oral pain), and functional capacity. This multidimensional assessment allows for the identification of specific nutritional deficits and supports the design of individualized interventions, making PG-SGA particularly valuable in oncology populations where tailored nutritional support is critical to improve treatment tolerance and patient outcomes.

In our study, nutritional assessment was performed systematically using the PG-SGA, which integrates both patient-reported symptoms and professional evaluation, and was further complemented with bioimpedance analysis to obtain objective data on body composition. This dual approach provided a comprehensive overview of patients’ nutritional status, enabling not only the detection of malnutrition but also the stratification of the level of intervention required. Incorporating both subjective and objective measures strengthens the diagnostic accuracy and underscores the importance of integrating standardized nutritional assessment into oncological practice from the beginning of chemotherapy, thereby facilitating early and individualized interventions.

### 4.5. Clinical and Strategic Implications

Our findings support the routine implementation of nutritional screening protocols in CRC patients, using validated tools and tailoring interventions to the toxicity profile of the chemotherapy regimen and the patient’s baseline characteristics. These strategies, framed within a multidisciplinary approach, have the potential to improve treatment tolerance, reduce morbidity, and preserve quality of life [13,26]. Furthermore, specific training of healthcare personnel in the management of oncological malnutrition is essential to improve early detection and optimize intervention.

### 4.6. Limitations

Although the sample size achieved allows us to draw relevant conclusions aligned with previous literature, having a larger cohort in future studies could facilitate the detection of smaller differences that may still hold clinical relevance. The single-center nature of the study, while ensuring homogeneity in procedures and diagnostic criteria, could benefit in future research from a multicenter approach that provides greater diversity of care settings. Likewise, the cross-sectional design, while appropriate for characterizing nutritional status at a given time, does not allow exploration of its evolution over time; therefore, future longitudinal studies would provide a more comprehensive perspective. Finally, although the present work focuses on clinical and nutritional variables, the inclusion of socioeconomic, lifestyle, and psychosocial determinants of nutrition—as well as direct measurement of quality-of-life outcomes—in upcoming studies could enrich the overall interpretation of results, since these factors can significantly influence nutritional risk and the effectiveness of interventions.

## 5. Conclusions

As demonstrated in the present study, there is a high nutritional risk in the evaluated oncology population, particularly relevant in patients with colorectal cancer undergoing chemotherapy. In our sample, patients treated with the FOLFOX regimen appear to present a higher nutritional risk, which may be related to the presence of more disabling symptoms during the last two weeks of treatment, significantly affecting intake and functional status. These results suggest that while FOLFOX is related to greater sensory impact and upper gastrointestinal symptoms, XELOX presents a profile more associated with lower digestive alterations and oral dryness.

The systematic application of nutritional screening from the beginning of treatment, using tools such as the PG-SGA and the GLIM criteria, is essential for early detection and individualized management, promoting better treatment tolerance and a positive impact on clinical outcomes and quality of life. Future multicenter and longitudinal studies will allow validation of these findings and optimization of nutritional support protocols in oncology.

## Figures and Tables

**Table 1:** Comparison of FOLFOX and XELOX chemotherapy regimens regarding nutrition-related disabling symptoms.

Symptoms the Patient Has Experienced That Have Prevented Them from Eating Enough over the Past 15 Days (PG-SGA)	FOLFOX (*p*-Value)	XELOX (*p*-Value)
Eating problems	**0.043**	0.931
Loss of appetite (3 pts.)	**0.041**	0.751
Constipation (1 pts.)	0.573	0.782
Canker sores (2 pts.)	0.749	0.229
Food tastes strange to me or doesn’t taste like anything (1 pts.)	**0.002**	0.533
I find smells unpleasant (1 pts.)	**0.021**	0.297
Pain (3 pts.)	0.153	0.114
Nausea (1 pts.)	**0.020**	0.521
Vomits (3 pts.)	0.709	0.719
Diarrhea (3 pts.)	0.150	**0.009**
Dry mouth (1 pts.)	0.482	**0.038**
Dysphagia (2 pts.)	0.475	0.077
I feel full after eating only a little (1 pts.)	0.337	0.271

*p*-values obtained through the χ^2^ test when administering FOLFOX and XELOX to the patients in the study sample, according to the symptomatology characteristic of a higher nutritional risk as assessed by the PG-SGA; Bold values that have been obtained with statistical significance (*p*-value < 0.05).

**Table 2:** Comparison of oxaliplatin and 5-fluorouracil/leucovorin chemotherapy regimens regarding nutrition-related disabling symptoms.

Symptoms the Patient Has Experienced That Have Prevented Them from Eating Enough over the Past 15 Days (PG-SGA)	OXALIPLATIN (*p*-Value)	5-FLUOROURACIL/LEUCOVORIN (*p*-Value)
Eating problems	0.206	**0.037**
Loss of appetite (3 pts.)	**0.034**	**0.019**
Constipation (1 pts.)	0.917	0.456
Canker sores (2 pts.)	0.276	0.517
Food tastes strange to me or doesn’t taste like anything (1 pts.)	0.112	**<** **0.001**
I find smells unpleasant (1 pts.)	**0.035**	**0.037**
Pain (3 pts.)	0.921	0.392
Nausea (1 pts.)	0.172	**0.006**
Vomits (3 pts.)	1	0.275
Diarrhea (3 pts.)	0.219	0.267
Dry mouth (1 pts.)	0.243	0.727
Dysphagia (2 pts.)	0.106	0.485
I feel full after eating only a little (1 pts.)	0.156	0.244

*p*-values obtained through the χ^2^ test when administering Oxaliplatin (XELOX), 5-Fluorouracil, and Leucovorin (FOLFOX) to the patients in the study sample, according to the symptomatology characteristic of a higher nutritional risk as assessed by the PG-SGA scale. Bold values that have been obtained with statistical significance (*p*-value < 0.05).

## Data Availability

The original contributions presented in this study are included in the article. The datasets generated and/or analyzed during the current study are available from the corresponding author upon reasonable request.

## Abbreviations

The following abbreviations are used in this manuscript:

CRC	Colorectal Cancer
PG-SGA	Patient-Generated Subjective Global Assessment
BMI	Body Mass Index
GLIM	Global Leadership Initiative on Malnutrition
MST	Malnutrition Screening Tool
MUST	Malnutrition Universal Screening Tool
